# A retrospective cohort study on the outcomes of ischemic stroke patients with adjuvant Korean Medicine treatment

**DOI:** 10.1038/s41598-018-19841-w

**Published:** 2018-01-26

**Authors:** Ye-Seul Lee, Seungwon Kwon, Younbyoung Chae, Bo-Hyoung Jang, Seong-Gyu Ko

**Affiliations:** 1 Acupuncture and Meridian Science Research Center, College of Korean Medicine, Kyung Hee University, Seoul, 02447 Republic of Korea; 2 Department of Cardiology and Neurology, College of Korean Medicine, Kyung Hee University, Seoul, 02447 Republic of Korea; 3 Department of Preventive Medicine, College of Korean Medicine, Kyung Hee University, Seoul, 02447 Republic of Korea

## Abstract

This study aims to examine the long-term effect of adjuvant treatment in Korean Medicine (KM) clinics in ischemic stroke patients, using a national sample cohort from the National Health Insurance Service in Korea between 2010 and 2013. The National Health Insurance Service – National Sample Cohort database from 2002 to 2013 was used in this study. Ischemic stroke patients were defined and covariates were included to account for socioeconomic variables, comorbidities and disease severity. Propensity score matching was applied. Cox proportional hazards modeling and Kaplan-Meier analysis were applied to determine the differences between KM and non-KM treated groups. The results shows that KM-treated group had a higher probability of survival than non-KM group patients. No significant difference was observed between the risk of readmission between the treated and non-treated patients. Kaplan-Meier analysis showed a weak effect of KM treatments in the patients with 8 to 28 days of hospital stay for a lower risk of recurrence than in non-treated patients. In conclusion, KM treatment of mild to moderate ischemic stroke patients has a mild effect on the survival probability of stroke. Its effect for improving long-term recurrence need to be explored in the future studies.

## Introduction

Stroke is the second leading cause of death worldwide^1,2^; additionally, stroke is the leading cause of death from a single organ disease in Korea^3^. The recurrence rate after an ischemic stroke with an uncertain cause has been reported to be above 20% during the first 2 years after the index event^4^. The main priority in treating patients after the first stroke is secondary stroke prevention^5–7^; therefore, a low-cost, safe, and effective means of managing post-stroke symptoms and preventing secondary stroke is required for stroke patients. The costs of stroke to the household as well as to society indicate the importance of stroke management^5^.

East Asian Medicine, including Korean Medicine (KM) and Traditional Chinese Medicine (TCM), has been widely used in countries in Asia for the treatment of stroke for many centuries^8^. The medical effectiveness of East Asian Medicine, including acupuncture and herbal medicines, has become a subject of study in the past few decades, although experimental and clinical data have shown limited results when evaluating the efficacy of East Asian Medicine for post-stroke treatment and management^9–12^. A number of studies have focused on the effectiveness of acupuncture in treating stroke patients and have reported mixed results^5,9,13–21^. Many possible mechanisms of acupuncture have been suggested for both treatment and post-stroke management^22–26^. KM, including acupuncture and herbal medicines, is still widely used among ischemic stroke patients as an adjuvant therapy for the prevention of secondary stroke and mortality^7^.

In this study, we investigated the risk of long-term mortality and the hospital readmission rate in mildly to moderately severe ischemic stroke patients treated with KM compared to those who did not receive KM treatments based on the National Health Insurance Service - National Sample Cohort (NHIS-NSC).

## Methods

### Data source

Data in the NHIS-NSC from 2010 and 2011, including a total of 1,011,725 patients, were analyzed in this study. The NHIS-NSC is a population-based cohort from 2002 to 2013 that was established by the NHIS in Korea^27^. The NHIS-NSC data provide personal information, demographics and medical treatment data based on National Health Insurance (NHI) claims from medical institutions to the NHIS from inpatient and outpatient clinic visits for each individual patient^27^. Specific information contained in the NHI claims data include principal and additional diagnoses, as well as hospitalization and outpatient treatment, dates of examinations, medical fees, details of medical services, prescribed medications, hospital codes, and patients’ sex and age; all information is accumulated and categorized based on the examination documented in the claims from the medical institutions^28^. The Korean Classification of Diseases, sixth revision (KCD-6), was used to code the diagnoses in the NHIS-NSC database and is based on the International Classification of Diseases, Tenth Revision, Clinical Modification (ICD-10-CM). For confidentiality and ethical considerations, the identification numbers of the individuals in the cohort were encrypted and transformed using a random number string. Written consent was not obtained from the study participants because the retrospective data used encrypted identification of the individuals. This study was given a formal waiver for the need for consent by the Institutional Review Board of Kyung Hee University.

### Study population

In this nationwide retrospective cohort study, the claims data of a cohort of 1,011,725 patients was used to define a study population of ischemic stroke patients who were diagnosed from January 2010 to December 2011. In the dual-medical license system of Korea, doctors of Western medicine and doctors of Korean medicine separately diagnose and treat patients in different clinics and hospitals. In addition, the two groups of doctors consult and in diagnosis and treatment of patients, allowing for collaboration. Both of the medical treatments are reimbursed by NHIS, which allows for cross-identification of patients who used only the Western Medicine and those who used both Western and Korean Medicine. Therefore, our dataset includes patients who visited KM clinics in addition to the standard care in the WM clinics, and those who only visited WM clinics, of which both clinics include qualified clinicians with certified license by the state and with qualified diagnosis skills. Subjects included patients above the age of 18 years, with an inpatient claims record and admission of one day or more under the main disease diagnosis of the KCD-6 code I63 (ischemic stroke). The admission day of each newly diagnosed patient was defined as the index date for data analysis. To exclude patients who had medical histories of stroke and to remove the possibility of immortal time bias, all records of reported symptoms from 2002 to 2009 were examined for any form of stroke (Supplementary data 1). To ensure the validity of the diagnosis of ischemic stroke in our study cohort, only patients with a neuroimaging record of computerized tomography (CT) scans, magnetic resonance imaging (MRI), or magnetic resonance angiogram (MRA) when they were diagnosed with ischemic stroke were included. In addition, only patients with a medication record of antithrombotic, anticoagulant, or antiplatelet therapy (Supplementary data 2) between one day before and after the index date were included in the final study cohort.

### Intervention

The NHIS claims record was examined using reimbursement records of treatment in KM clinics after the index date of the ischemic stroke. Only the patients with treatment records in KM clinics after the discharge from the medical institution in which they were initially diagnosed with ischemic stroke and hospitalized were included in the cohort. The patients in the treated group received at least 3 treatment sessions, and the treatments included acupuncture, electroacupuncture, moxibustion, cupping therapies, and herbal complexes. Table 1 outlines the types of treatments and medical care that the KM-treated patients received. Only KM doctors with certified medical licenses are qualified to file NHIS claims for acupuncture treatment reimbursement; thus, the quality of acupuncture treatment in the medical institutions was indirectly controlled. Patients with and without treatments from KM clinics were followed from the index event until the end of 2013 to determine mortality as well as hospital readmission due to secondary stroke.

**Table 1:** Types of treatments received by KM-treated patients (n = 194) among ischemic stroke study cohort.

	Number of patients	%
Acupuncture	194	100
Electroacupuncture	180	92.8
Moxibustion	98	50.6
Cupping	122	62.9
Herbal medicine	75	38.7

### Outcomes

The long-term outcomes in this study included all-cause mortality and readmission due to any type of secondary stroke for more than 1 day, and with a neuroimaging record of computerized tomography (CT) scans, magnetic resonance imaging (MRI), or magnetic resonance angiogram (MRA). The NHIS-NSC cohort provided data on mortality as well as the admission records of all the patients included in the study.

### Stroke severity

To assess the stroke severity of the ischemic stroke patients in the study cohort, surgery records (Supplementary data 3), Charles Comorbidity Index (CCI), bacterial sensitivity test, nasogastric intubation, urinary catheterization, intensive care unit (ICU) stay, and manual ventilation on the first diagnosis of the ischemic stroke were collected. To assess the underlying comorbidities of ischemic stroke, diseases including hypertension, diabetes, hyperlipidemia, myocardial infarction, and renal failure were identified as the key stroke risk factors that might contribute to stroke recurrence and compound the effectiveness of the acupuncture treatment^1,2,4,29,30^. Furthermore, anticoagulation therapy and antiplatelet therapy, as well as the types of medical institutions in which the patient was hospitalized were also assessed^3,7^. The length of stay upon the first admission was assessed by division into three categories: 7 days or less, between 8 and 28 days, and 29 days or longer. In further data analysis, we only included patients with a length of stay for 28 days or less to include patients with mild to moderately severe stroke symptoms and exclude patients who had limited access to KM treatments as a result of lower functional independence^31,32^.

### Statistical analysis

A propensity score was calculated for each patient based on key baseline covariates with a known association with KM treatments or the outcomes in the study, including age, sex, economic status, disease severity according to the types of medical institutions, length of stay, and types of medication were also included in PSM calculation using the “nearest” method. We tested for difference-in-means of the variables between KM and non-KM groups before finalizing the selection of cohorts. We also did a correlation analysis on the corresponding set of scaled Schoenfeld residuals with time to test for independence between residuals and time. The resulted cohort which did not show differences in means of the variables was selected for further analysis.

Chi-square tests were used to compare the distributions of age, sex, economic status, comorbidities, types of medical institutions, length of stay, CCI, types of medications, and medical interventions between the groups with and without KM treatment. To eliminate the risk of immortal time bias, the KM treatment group’s observation period was adjusted to the time when the patient first visited the KM clinic. The Cox proportional hazards ratio was applied to calculate the hazard ratios (HRs) of mortality and readmission with 95% confidence intervals (CIs). Kaplan-Meier curve analysis was used to measure mortality over time, and the cumulative hazard curve was applied to measure readmission over time. In the Kaplan-Meier curve analysis of both mortality and readmission, patients were divided into subgroups by the length of hospital stay and medication to control for stroke severity. All statistical analyses were conducted using “MedicalRisk”, “ICD”, “survival” and “survsim” package in R software (version 3.2.3 “Wooden Christmas-Tree”, http://r-project.org/), with *p* < 0.05 representing statistical significance.

## Results

### Baseline statistics

The final population of mild to moderately severe ischemic stroke patients from 2010 and 2011 (n = 448) was divided into two groups including those with (n = 108) or without (n = 340) adjuvant treatment from KM clinics according to the claims data. The cohort for survival analysis was developed with mild cases of ischemic stroke consisted of patients who stayed in the hospital for 8 to 28 days. Furthermore, as the non-KM treated group of mild ischemic stroke did not receive anticoagulant therapies, we also excluded this medication from the inclusion criteria of KM-treated group of patients. The resulted cohort (n = 194) was created from ischemic stroke patients between 2010 and 2011 with and without adjuvant treatment in KM clinics, from the index date to the last day of 2013. After the matching analysis by propensity scoring, no significant difference was found in socio-demographic factors, underlying comorbidities, length of stay, CCI, manual ventilation, nasogastric intubation, bacterial antibiotic sensitivity test, urinary catheterization, ICU stay, and the types of medication during hospitalized care during the first ischemic stroke, or the type of hospitalized medical institutions between the ischemic stroke patients with and without acupuncture treatment (Table 2).

**Table 2:** Baseline characteristics of propensity score-matched ischemic stroke study cohort with and without KM treatments.

		non-KM treated (n = 372)	%	KM-treated (n = 372)	%	*P*-value
Age	20–29	0	0%	1	1%	0.910
	30–39	2	2%	3	3%	
	40–49	9	9%	9	9%	
	50–59	15	15%	15	15%	
	60–69	27	28%	28	29%	
	70–79	30	31%	27	28%	
	80–	14	14%	14	14%	
Sex	Male	56	58%	58	60%	0.884
	Female	41	42%	39	40%	
Economic	Very Low	27	28%	28	29%	0.783
	Low	12	12%	11	11%	
	Moderate	14	14%	19	20%	
	High	16	16%	15	15%	
	Very High	28	29%	24	25%	
Comorbidities	Diabetes	45	46%	54	56%	0.9745
	Hypertension	60	62%	60	62%	
	Hyperlipidemia	54	56%	49	51%	
	Myocardial infarction	2	2%	2	2%	
	Renal failure	2	2%	1	1%	
CCI	0	95	98%	95	98%	1.000
	1	0	0%	0	0%	
	2	2	2%	2	2%	
Inpatient days	0–7	37	38%	37	38%	1.000
	8–29 days	60	62%	60	62%	
Medical institution types	Tertiary Hospital	87	90%	87	90%	1.000
	General Hospital	10	10%	10	10%	
Medication	Anticoagulants	0	0%	0	0%	1.000
	Antiplatelets	97	100%	97	100%	
Medical intervention	Bacterial antibiotics sensitivity test	8	8%	8	8%	0.857
	Manual ventilation	0	0%	0	0%	
	Nasogastric intubation	0	0%	0	0%	
	Urinary Catheterization	22	23%	24	25%	
	ICU stay	14	14%	18	19%	

### Survival

During a mean follow-up time of 36.2 weeks (range: 0–200 weeks), a total of 35 patients (18%, 17 patients in KM cohort and 18 patients in non-KM cohort) died, and 148 patients (76.3%) were readmitted to the hospital for more than one day. The mortality rate was 68.04 per 1,000 population per year in KM cohort, and 47.27 per 1,000 population per year in non-KM cohort. The crude and adjusted propensity score-matched HRs for post-stroke all-cause mortality and readmissions due to secondary stroke are shown in Table 3. Patients who received KM treatment had a lower long-term risk of all-cause mortality with an adjusted propensity score-matched HR of 0.44 (95% CI, 0.21–0.93). The long-term risk of readmission did not differ significantly between patients with and without KM treatment (adjusted HR, 1.02; 95% CI, 0.73–1.43 and adjusted HR, 1.01; 95% CI, 0.30–3.38, respectively).

**Table 3:** Hazard ratios of all-cause mortality and readmission in propensity score-matched ischemic stroke study cohort with and without KM treatments.

	Crude HR (95% CI)	*P*-value	Adjusted HR	*P*-value
Mortality	0.44 (0.21–0.93)	0.03*	0.55 (0.28–1.10)*	0.09
Readmission	1.02 (0.73–1.43)	0.92	0.87 (0.62–1.20)	0.39

**Figure 1:**
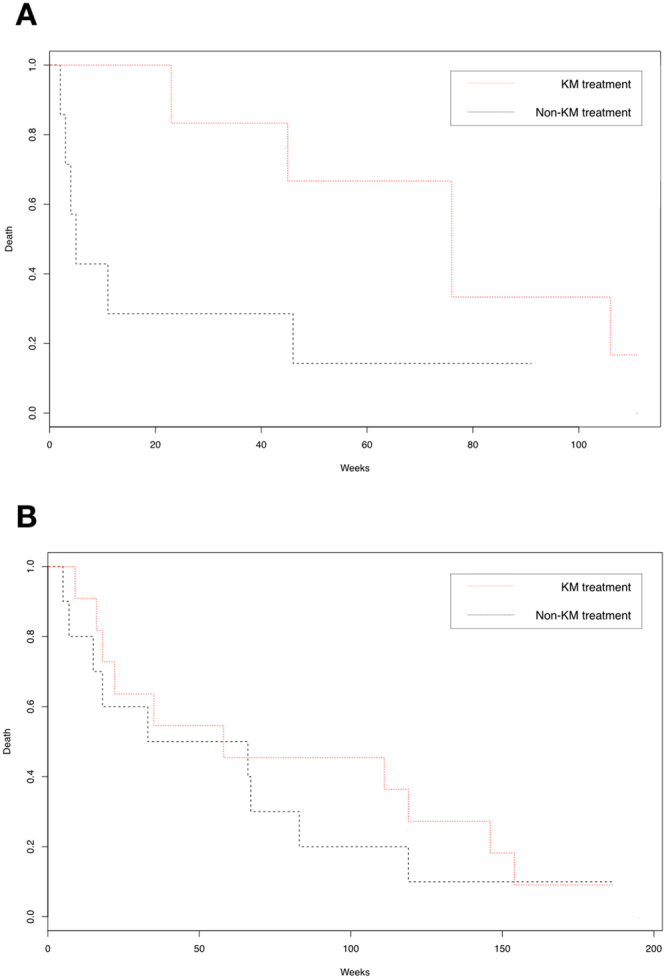
Estimated all-cause survival probability (%) for ischemic stroke patient cohort with and without treatments in Korean Medical clinics. Figure 1A: Survival probability of the patient cohort with length of stay for less than 8 days. (Log tank test, p = 0.02). Figure 1B: Survival probability of the patient cohort with length of stay for 8 to 28 days. (p = 0.70). X-axis: time of survival in weeks; Y-axis: estimated survival probability (%). Red line: patients who received KM treatments; Black line: patients who did not receive KM treatments.

**Figure 2:**
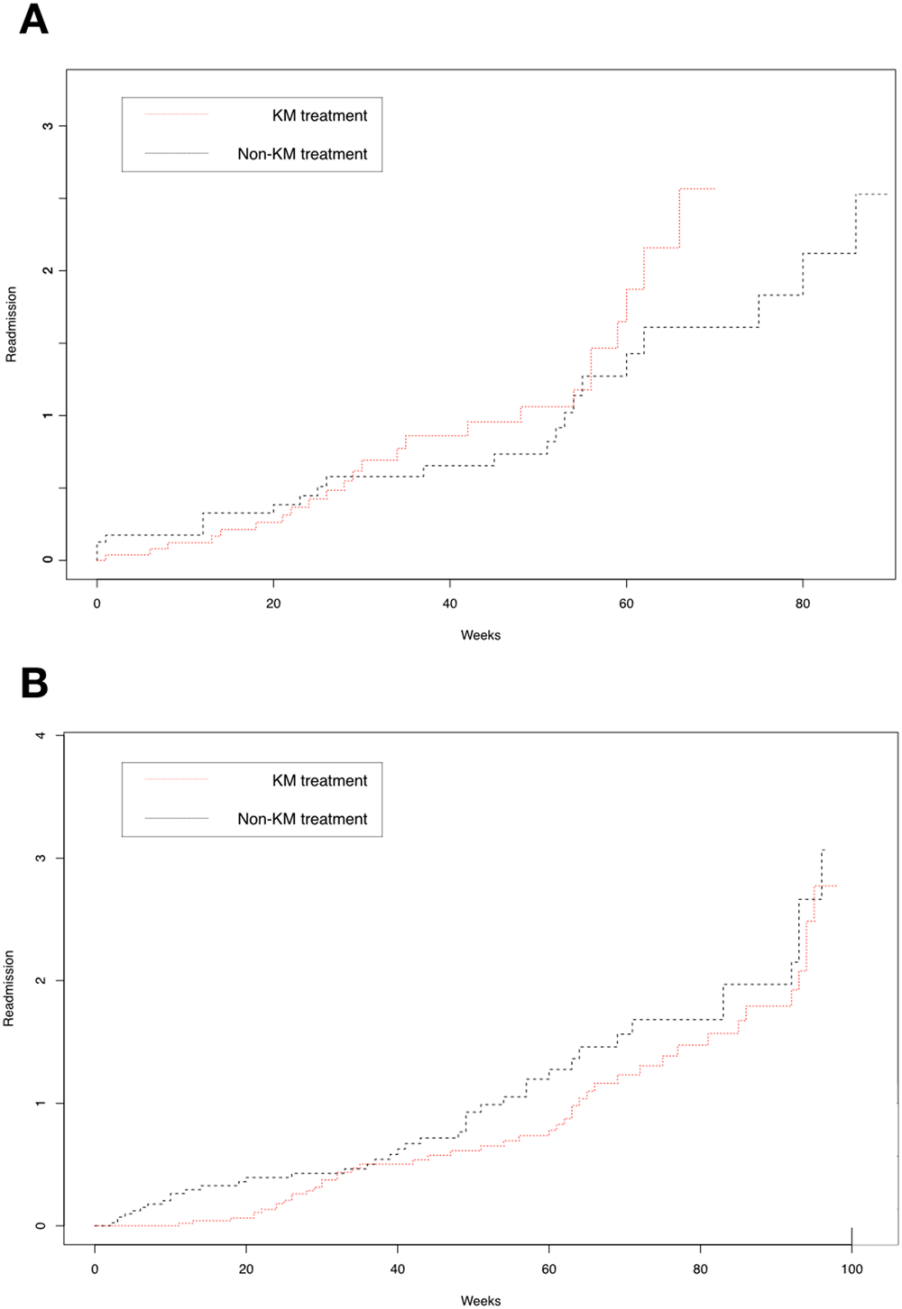
Cumulative risk of readmission (%) for ischemic stroke patient cohort with and without treatments in Korean Medical clinics Fig. 2A: Readmission risk of the patient cohort who stayed in the hospital for less than 8 days (p = 0.60). Figure 2B: Readmission risk of the patient cohort who stayed in the hospital for 8 to 28 days (p < 0.001). X-axis: time of survival in weeks; Y-axis: cumulative risk of readmission (%). Red line: patients who received KM treatments; Black line: patients who did not receive KM treatments.

Kaplan-Meier analysis showed that among the patients who stayed in the hospital for less than 8 days, patients in the non-KM treated group had higher mortality (log rank test, p = 0.02; Fig. 1A). Among the patients who stayed in the hospital for 8 to 28 days, KM and non-KM treated groups did not show any differences (Fig. 1B). In the case of readmission, KM and non-KM treated groups of the patients who stayed in the hospital for less than 8 days had no difference in the risk of readmission (Fig. 2A). Readmission of the patients who stayed in the hospital for 8 to 28 days also did not show any significant difference (Fig. 2B).

## Discussion

This study assessed the propensity score-matched risk of the long-term outcomes of all-cause mortality and recurrence, in mild to moderately severe ischemic stroke patients using the NHIS-NSC. The results of the Cox proportional hazards model showed a higher survival probability among patients treated in KM clinics than in non-treated patients, and no significant difference in the risk of hospital readmission was found among the treated and non-treated patients. Among the patients who did not stay for longer than one week after the onset of ischemic stroke, the KM-treated patients showed higher probability of survival. On the other hand, there were no significant differences of survival between KM and non-KM treated patients in the patient cohort who stayed for 8 to 28 days in the hospital. The analysis has been thoroughly controlled for the possibility of selection bias, and thus might imply the effect of Korean Medicine in the long-term survival of mild ischemic stroke patients. However, the cases of more severe patients who stayed for shorter period of time in the hospital have also been observed in previous studies^33,34^, and therefore, caution is required in the interpretation of this result. On the other hand, weak but consistent tendencies of KM treatment effects were observed in both mortality and recurrence of ischemic stroke, and the statistical significance may have been limited due to the conservative analytical approach taken in our study in creating the cohort and proportional hazards modeling.

This observational study took a large effort in minimizing selection bias and may further explain the heterogeneous results from previous studies. First, unlike previous studies that only employed the disease codes to define stroke^20^, this study defined newly diagnosed ischemic stroke patients in 2010 and 2011 through their neuroimaging records, the length of stay for the first ischemic stroke, and the medical prescriptions of antiplatelet and antithrombotic therapies in the claims data from one day before to one day after the index date. In addition, the medical histories from 2002 to the day before the index date were thoroughly examined for any record of cerebral disease. Second, while the severity of the stroke, in addition to socioeconomic variables, was not sufficiently accounted for in the previous studies, the severity of the patients was taken into account through their Charlson comorbidity index and underlying comorbidities such as hypertension, diabetes, and hypercholesterolemia, the risk factors of stroke^3^, the length of stay during their first stroke^32^, and the type of medical institution in which they were hospitalized^29–31^. We also analyzed the stroke severity through the records of medical interventions including length of stay, ICU stay, bacterial antibiotics sensitivity test, urinary catheterization, nasogastric intubation, and manual ventilation. Our study analyzed only mild to moderately severe ischemic stroke patients to include patients with sufficient functional independence to access adjuvant KM treatments^35,36^.

The observed effects of KM treatments, including acupuncture, in all sessions in this study are consistent with two recent randomized trials with long-term follow-up periods^9,37^. While a limited improvement of leg function^37^ and a tendency of reduced mortality and functional dependency was observed in acupuncture-treated patients^9^, limited evidence and a remaining demand for a larger randomized trial with long-term observation of the effectiveness of acupuncture after stroke still exist. However, the exact mechanisms responsible for the possible lower risk of mortality of after KM treatment remain to be clarified^21^. Previous studies have suggested that the salutary actions of acupuncture affect vascular function in ischemic stroke patients^22–25^. Pretreatment using electroacupuncture may reduce the size of an infarct through regulation of the endocannabinoid system and improve the neurological outcome^26^. Therapeutic aid by acupuncture in some stroke patients may increase physical activity and reduce stroke recurrence risk, in addition to improving the rehabilitation outcome^38,39^. Despite the insufficient explanation of the mechanism of acupuncture in stroke patients and the lack of solid evidence regarding its clinical utility^13^, several recent studies have shown some improvement in stroke outcome through acupuncture treatment^9,13^. On the other hand, herbal complexes have been suggested as useful agents for various issues such as recovery of motor function^40–42^, sensory disturbances^43^, emotional problems^44^, cognitive dysfunction^45^, aphasia^46^ and prevention of recurrence^47^. The assumed mechanism of herbal medicines in stroke is an anti-inflammatory effect on neuro-inflammation^48^. Furthermore, previous studies suggest that herbal medicines have antihypertensive^49^ and antilipidemic effects.

This study has several limitations. First, the retrospective cohort did not include detailed information on the biochemical measures, including clinical features of stroke such as the location of specific lesions and factors related to lifestyles. The types of different medications and their possible implications in the post-stroke quality of life need to be assessed in the future as well. In addition, the combination of treatments received in the KM clinics vary among patients, which only allows to estimate a general effect of KM treatments. We also cannot exclude that the results from this study are influenced by confounding factors due to the observational nature of our study design; furthermore, the risk of selection bias as well as the limits of propensity score matching can’t be overlooked^50^. However, the selection of eligible patients with a length of stay of 29 days or less, as well as patients among this group who received treatments in KM clinics, was performed from a cohort of 1,011,725 patients which reduces the risk of selection bias. In addition, covariates based on previous were included in setting the propensity score; and the difference-in-means were tested for each covariate between groups with and without KM treatments to minimize the risk of confounding factors through a combination of restriction, propensity score matching, and multivariable regression^50,51^.

In conclusion, this study assessed the efficacy of KM treatments including acupuncture and herbal medicines that were used as an adjunct to standard care in post-stroke management. While the results need to be interpreted with caution, KM-treated patients showed higher survival probability of stroke, and although insignificant, a weak tendency of reduced long-term recurrence risk was observed. Although the result does not fully support the utilization of KM to prevent readmission or recurrence of secondary stroke, it may have an effect on the overall survival of ischemic stroke patients. Large-scale clinical trials with long-term observations are needed to confirm the potential benefit of KM treatments and acupuncture for post-stroke management and rehabilitation.

### Disclosure

The funders had no role in the study design, data collection and analysis, or the decision to publish the paper.

## Electronic supplementary material

Dataset 1

## References

[1] Donnan GA, Fisher M, Macleod M, Davis SM (2008). Stroke. Lancet.

[2] Go AS (2013). Executive summary: heart disease and stroke statistics–2013 update: a report from the American Heart Association. Circulation.

[3] Hong KS (2013). Stroke statistics in Korea: part I. Epidemiology and risk factors: a report from the korean stroke society and clinical research center for stroke. J Stroke.

[4] Petty GW (2000). Ischemic stroke subtypes: a population-based study of functional outcome, survival, and recurrence. Stroke.

[5] Shih CC (2015). A Retrospective Cohort Study Comparing Stroke Recurrence Rate in Ischemic Stroke Patients With and Without Acupuncture Treatment. Medicine (Baltimore).

[6] Fonarow GC (2010). Characteristics, performance measures, and in-hospital outcomes of the first one million stroke and transient ischemic attack admissions in get with the guidelines-stroke. Circ Cardiovasc Qual Outcomes.

[7] Hong KS (2013). Stroke Statistics in Korea: Part II Stroke Awareness and Acute Stroke Care, A Report from the Korean Stroke Society and Clinical Research Center For Stroke. J Stroke.

[8] Pandian JD, Liu M, Misbach J, Venketasubramanian N (2011). Alternative therapies for stroke treatment in Asia. Int J Stroke.

[9] Zhang S (2015). Acupuncture efficacy on ischemic stroke recovery: multicenter randomized controlled trial in China. Stroke.

[10] Wu B (2007). Meta-analysis of traditional Chinese patent medicine for ischemic stroke. Stroke.

[11] Chen CL (2013). Chinese medicine neuroaid efficacy on stroke recovery: a double-blind, placebo-controlled, randomized study. Stroke.

[12] Wu, B. & Liu, M. How to improve the quality of a clinical trial on traditional Chinese medicine for stroke. *Stroke* **40**, e641–642; author reply e643–644, 10.1161/STROKEAHA.109.563072 (2009). 10.1161/STROKEAHA.109.563072 19762693

[13] Wu H (2008). Acupuncture for stroke rehabilitation. Stroke.

[14] Wei YC (2011). Pilot scheme of health policy in stroke adjuvant acupuncture therapy for acute and subacute ischemic stroke in taiwan. Evid Based Complement Alternat Med.

[15] Hawk C, Ndetan H, Evans MW (2012). Potential role of complementary and alternative health care providers in chronic disease prevention and health promotion: an analysis of National Health Interview Survey data. Prev Med.

[16] Sze FK, Wong E, Yi X, Woo J (2002). Does acupuncture have additional value to standard poststroke motor rehabilitation?. Stroke.

[17] Johansson BB (2001). Acupuncture and transcutaneous nerve stimulation in stroke rehabilitation: a randomized, controlled trial. Stroke.

[18] Wu P, Mills E, Moher D, Seely D (2010). Acupuncture in poststroke rehabilitation: a systematic review and meta-analysis of randomized trials. Stroke.

[19] Chen L (2014). Acupuncture for acute stroke: study protocol for a multicenter, randomized, controlled trial. Trials.

[20] Chang CC (2016). Outcomes after stroke in patients receiving adjuvant therapy with traditional Chinese medicine: A nationwide matched interventional cohort study. J Ethnopharmacol.

[21] Lu CY (2017). Acupuncture Therapy and Incidence of Depression After Stroke. Stroke.

[22] Flachskampf FA (2007). Randomized trial of acupuncture to lower blood pressure. Circulation.

[23] Kim DD, Pica AM, Duran RG, Duran WN (2006). Acupuncture reduces experimental renovascular hypertension through mechanisms involving nitric oxide synthases. Microcirculation.

[24] Tsuchiya M, Sato EF, Inoue M, Asada A (2007). Acupuncture enhances generation of nitric oxide and increases local circulation. Anesth Analg.

[25] Hsieh CH (2010). The effects of auricular acupressure on weight loss and serum lipid levels in overweight adolescents. Am J Chin Med.

[26] Wang Q (2009). Pretreatment with electroacupuncture induces rapid tolerance to focal cerebral ischemia through regulation of endocannabinoid system. Stroke.

[27] Lee, J., Lee, J. S., Park, S. H., Shin, S. A. & Kim, K. Cohort Profile: The National Health Insurance Service-National Sample Cohort (NHIS-NSC), South Korea. *Int J Epidemiol*, 10.1093/ije/dyv319 (2016). 10.1093/ije/dyv319 26822938

[28] Jee SH (2008). Stroke risk prediction model: a risk profile from the Korean study. Atherosclerosis.

[29] Heuschmann PU (2004). Predictors of in-hospital mortality and attributable risks of death after ischemic stroke: the German Stroke Registers Study Group. Arch Intern Med.

[30] Katzan IL, Dawson NV, Thomas CL, Votruba ME, Cebul RD (2007). The cost of pneumonia after acute stroke. Neurology.

[31] Ingeman A, Andersen G, Hundborg HH, Svendsen ML, Johnsen SP (2011). In-hospital medical complications, length of stay, and mortality among stroke unit patients. Stroke.

[32] Saxena SK, Ng TP, Yong D, Fong NP, Gerald K (2006). Total direct cost, length of hospital stay, institutional discharges and their determinants from rehabilitation settings in stroke patients. Acta Neurol Scand.

[33] Heuschmann PU (2004). Predictors of in-hospital mortality in patients with acute ischemic stroke treated with thrombolytic therapy. JAMA.

[34] Dewan KR, Rana PV (2014). A study of seven day mortality in acute ischemic stroke in a teaching hospital in Chitwan. J Nepal Health Res Counc.

[35] Tan WS, Heng BH, Chua KS, Chan KF (2009). Factors predicting inpatient rehabilitation length of stay of acute stroke patients in Singapore. Arch Phys Med Rehabil.

[36] Toh HJ, Lim ZY, Yap P, Tang T (2017). Factors associated with prolonged length of stay in older patients. Singapore Med J.

[37] Park J (2005). Acupuncture for subacute stroke rehabilitation: a Sham-controlled, subject- and assessor-blind, randomized trial. Arch Intern Med.

[38] Wen CP (2011). Minimum amount of physical activity for reduced mortality and extended life expectancy: a prospective cohort study. Lancet.

[39] Boysen G, Krarup LH (2009). Benefits of physical activity for stroke survivors. Expert Rev Neurother.

[40] Goto H (2011). A chinese herbal medicine, tokishakuyakusan, reduces the worsening of impairments and independence after stroke: a 1-year randomized, controlled trial. Evid Based Complement Alternat Med.

[41] Yu M (2015). The beneficial effects of the herbal medicine Di-huang-yin-zi (DHYZ) on patients with ischemic stroke: A Randomized, Placebo controlled clinical study. Complement Ther Med.

[42] Xu JH (2015). Wen Dan Decoction for hemorrhagic stroke and ischemic stroke. Complement Ther Med.

[43] Jung WS, Moon SK, Park SU, Ko CN, Cho KH (2004). Clinical assessment of usefulness, effectiveness and safety of jackyakamcho-tang (shaoyaogancao-tang) on muscle spasm and pain: a case series. Am J Chin Med.

[44] Yun SP (2007). Hwangryunhaedogtang (huanglianjiedutang) treatment for pathological laughter after stroke and importance of patterns identification: a preliminary study. Am J Chin Med.

[45] Zeng L (2015). Can Chinese Herbal Medicine Adjunctive Therapy Improve Outcomes of Senile Vascular Dementia? Systematic Review with Meta-analysis of Clinical Trials. Phytother Res.

[46] Jung W, Kwon S, Park S, Moon S (2012). Can combination therapy of conventional and oriental medicine improve poststroke aphasia? Comparative, observational, pragmatic study. Evid Based Complement Alternat Med.

[47] Cho K (2008). A preliminary study on the inhibitory effect of Chunghyul-dan on stroke recurrence in patients with small vessel disease. Neurol Res.

[48] Gu Y, Chen J, Shen J (2014). Herbal medicines for ischemic stroke: combating inflammation as therapeutic targets. J Neuroimmune Pharmacol.

[49] Cao Y, Liu LT, Wu M (2017). Is Chinese herbal medicine effective for elderly isolated systolic hypertension? A systematic review and meta-analysis. Chin J Integr Med.

[50] Sainani KL (2012). Propensity scores: uses and limitations. PM R.

[51] Austin PC (2009). Balance diagnostics for comparing the distribution of baseline covariates between treatment groups in propensity-score matched samples. Stat Med.

