# Supplementary material for: A retrospective cohort study on the outcomes of ischemic stroke patients with adjuvant Korean Medicine treatment
Source: Sci Rep. 2018 Jan 26;8:1656. doi: 10.1038/s41598-018-19841-w (PMC5786017; doi:10.1038/s41598-018-19841-w)
Supplement: Supplementary file 1 — Dataset 1 [file 41598_2018_19841_MOESM1_ESM.doc]

*Submission to Scientific Reports*

**A retrospective cohort study on the outcomes of ischemic stroke patients with adjuvant Korean Medicine treatment**

Ye-Seul Lee^1^, Seungwon Kwon^2^, Younbyoung Chae^1^, Bo-Hyoung Jang^3^, Seong-Gyu Ko^3^

1. Acupuncture and Meridian Science Research Center, College of Korean Medicine,
Kyung Hee University, Seoul, 02447, Republic of Korea

2. Department of Cardiology and Neurology, College of Korean Medicine, Kyung Hee University, Seoul, 02447, Republic of Korea

3. Department of Preventive Medicine, College of Korean Medicine, Kyung Hee University, Seoul 02447, Republic of Korea

The authors have declared that no competing interests exist. The funders had no role in study design, data collection and analysis, decision to publish, or preparation of the manuscript.

Word count: 4,592

Tables: 3

Figures: 2

Ye-Seul Lee, MD (DKM), MPH

Acupuncture and Meridian Science Research Center, College of Korean Medicine, Kyung Hee University, Seoul, 02447, Korea

[jparadise.lys@gmail.com](mailto:jparadise.lys@gmail.com)

Seungwon Kwon, MD (DKM), PhD

Department of Cardiology and Neurology, College of Korean Medicine, Kyung Hee University, Seoul, 02447, Republic of Korea

[kmdkwon@gmail.com](mailto:kmdkwon@gmail.com)

Younbyoung Chae, MD (DKM), PhD

Acupuncture and Meridian Science Research Center, College of Korean Medicine, Kyung Hee University, Seoul, 02447, Korea

[ybchae@khu.ac.kr](mailto:ybchae@khu.ac.kr)

Seong-Gyu Ko, MD (DKM), MPH, PhD

Department of Preventive Medicine, College of Korean Medicine, Kyung Hee University,

Seoul, 02447, Korea

[epiko@khu.ac.kr](mailto:epiko@khu.ac.kr)

Corresponding author: Bo-Hyoung Jang, MD (DKM), MPH, PhD

Department of Preventive Medicine, College of Korean Medicine, Kyung Hee University,

Seoul, 02447, Korea

Tel: +82-2-961-9278

Fax: +82-2-966-1165

E-mail: [bhjang@khu.ac.kr](mailto:bhjang@khu.ac.kr)

**Supplementary data 1**

Any form of stroke history from 2002 to the index date included the following disease codes.

(I60: Nontraumatic subarachnoid hemorrhage; I61: Nontraumatic intracerebral hemorrhage; I62: Other and unspecified nontraumatic intracranial hemorrhage; I63: Cerebral infarction; I69: Sequelae of cerebrovascular disease; G46: Vascular syndromes of the brain in cerebrovascular diseases)

**Supplementary data 2**

Examined prescriptions

|  | **Examined prescriptions** |
| --- | --- |
| **antithrombotic, anticoagulant, or antiplatelet therapy**  (Alphabetical order) | apixaban, argatroban, aspirin, clopidogrel, cilostazol, dabigatran, dalteparin, enoxaparin, heparin, ibudilast, indobufen, mesoglycan, mesoglycan sodium, nadroparin, ozagrel sodium, rivaroxaban, sulodexide, sulfomucopolysaccharide, ticlopidine, triflusal, warfarin |

**Supplementary data 3**

| **Examined surgery records** |
| --- |
| craniotomy, ventriculostomy with shunting for hemorrhagic disease, tracheostomy |
